# Feminist Constellations in Legal History

**DOI:** 10.1093/ojls/gqag009

**Published:** 2026-03-10

**Authors:** Erika Rackley, Sharon Thompson

**Keywords:** activism, constellations, feminism, legal history, social justice, women’s history

## Abstract

Astronomers use constellations—groups of named patterns or sets of stars—to map the galaxies around us. More than simply maps of the sky, for centuries constellations and stars within them have enabled us to look into the past, as well as to navigate pathways into the future. So much for space, but what about law? What if legal historians were to look to the constellations? What if legal historians were to apply the tools and techniques of astronomers to feminists’ relationship with law and law reform? What might feminist legal constellations look like? And how might they inform our understandings of law and feminist activism? In this article we outline a new methodology: a feminist constellations approach to legal history. We argue that if feminist legal history is to be truly transformative, it needs to be bolder, and to go further than it does at present. And to do this, it needs to locate feminists and feminist endeavour within feminist constellations in law.

## 1. Introduction

*Constellation, n.: A number of fixed stars grouped together within the outline of an imaginary figure traced on the face of the sky.* ^1^

Speaking in 2017 about her experiences at the Greenham Common Women’s Peace Camp, feminist activist Di McDonald commented that ‘At Greenham, everyone was equal, and everyone had the opportunity to speak or not speak in a meeting, because there were no leaders. We had a saying: “The only stars are in the sky”.’^2^ As the women of Greenham Common knew, great women—and men—rarely act alone. Ahead, behind and beside every heroine and hero sits a network of women and men. Women and men who said they *could*, who inspired, encouraged and supported their rebellious activities and who facilitated and shaped their heroic credentials. For feminist scholars and historians, these friendships, networks and collaborations are essential to understanding the lives of ‘great’ women and men, and feminist history more broadly.^3^ So too feminist *legal* history. After all, as Rackley and Auchmuty and others have argued, feminist legal history is not simply about identifying heroines or uncovering ‘forgotten’ women and adding them to the existing plotlines—though this is important. Nor is ‘feminist’ a synonym for ‘women’s history’—legal or otherwise.^4^ In fact, feminist legal history is not really *about* women at all (though they play a large part in its substantive content). Rather, feminist legal history—as with feminism more broadly—is about power and relationships. It is about who has power and who does not, and why this is so. It is about the interconnections not only between and among women and women’s organisations and groups—their commonalities and, at times significant, disagreements—but also the interaction between women, men and those who live outside and resist such binaries. This includes women’s engagement with men who facilitated women’s campaigns and those who resisted, men and women who empowered women and those who did not.^5^

And yet, while UK scholarship exploring the substantive aims of feminist legal history abounds,^6^ far less has explored its methodological and political objectives, that is, *how* feminists in law are recovered and why this is important.^7^ At the same time, despite, rightly, far greater recognition that ‘women do legal history too’, there has been relatively little discussion as to whether they might do it for different reasons. Put another way, to date, the focus has been on what feminism might bring to the discipline of legal history, rather than on what legal history might bring to feminism. Or, more specifically, what *feminist* legal history might bring—and, vitally, how it might be brought—to current social justice campaigns.

This article seeks to address that gap. Building on existing feminist, feminist legal history and history methodologies,^8^ and taking inspiration from the women of Greenham Common’s invocation of the stars in the sky as our starting point, we make the case for a new approach to feminist legal history: a feminist constellations approach. Drawing on the tools and techniques of astronomers, we argue that, just as farmers, explorers and storytellers have turned to the constellations as a way of providing a meaning or explanation for earthly happenings, the language of constellations provides us with a means through which to identify, challenge and expand our understanding of the relationships between, across and beyond women’s individual lived experiences and their collective endeavours, aspirations and campaigning.^9^ In this way, our approach is feminist in every sense: substantively, by seeking to identify and nurture feminist engagement with how law has been reformed, and methodologically, by using a distinctly feminist lens to present a political challenge to dominant legal histories that have marginalised women’s role. We identify five characteristics of a feminist constellations approach: (i) the recognition and embrace of multiple temporalities; (ii) the making visible of neglected continuities; (iii) the amplification of inconspicuous success and impact; (iv) a willingness to engage in the process of star hopping—that is, of using brighter stars to find others—across and within (dark) constellations; and (v) the use of the feminist constellations as a system of navigation. Taking each in turn, we argue that a feminist constellations approach allows us to challenge established authorities and canons to reveal the hidden and neglected, and to amplify the multidimensional, complex and troubling. We argue that, just as the celestial constellations are used by explorers to plot their way forward, a feminist constellations approach is not just about how we write histories of the past, but how we campaign in the present and future. A feminist constellations approach to legal history seeks to place legal history at the heart of contemporary feminist projects. This is important, for if feminist legal history is to live up to its transformative aspirations^10^ —that is, if it is to be about more than recovering silenced or ‘lost’ voices, if it is to speak to the future and effect political change—then feminist legal historians need to be bolder, and to go further than they do at present. We need to move beyond the simple recovery of history and to speak to the (re)construction of feminist presents and futures. And to do this, we need not only to locate women and feminist endeavours ‘within complex and multiple temporalities’,^11^ that is, across time, place, periods and jurisdictions, but also to use these feminist constellations in law as a means of navigating the past, while informing present and future social justice and feminist campaigns.

## 2. Looking to the Sky: Stars, Constellations and Asterisms

A feminist constellations approach to legal history borrows the insights of astronomers as a means of mapping, challenging and resisting the structure and power dynamics of the legal universe through the mapping of feminist constellations. It is necessary, therefore, to begin with a brief look at the tools astronomers have used to understand space to establish the foundations of the characteristics of the feminist constellations approach that follow. Here, we focus on three: constellations, asterisms and bright stars.

### A. Constellations

Ever since humans first looked to the sky, they have used the location of the sun, moon and stars as a means of survival. In every culture, astrological methods have been used to develop tools and calendars to help us find our way and structure our lives. Constellations—groups of named patterns or sets of stars—are a vital part of this. For centuries, these patterns, and the names given to them, have varied. This makes sense. After all, only people who have seen emus or llamas will ‘see’ them ‘in an arbitrary pattern in the sky’.^12^ However, since the early 1900s, there has been an internationally agreed set of 88 constellations spanning the southern and northern hemispheres and contained within officially defined boundaries.^13^ Alongside this ‘official’ or agreed map of the sky, other ancient^14^ and indigenous star maps continue to be used, challenging the dominance and ‘authority’ of the westernised, ‘Greek systems’ of constellations and the assumption that there is only one map of the sky rather than a series of maps each ‘filled with the bias’, history and culture ‘of the map-maker’.^15^ The bright star Betelgeuse, for example, within the official constellation of Orion, is also part of at least 17 other constellations around the world, including First Slim One (Navajo), Buffalo Embryo (Dakota), The Sticks (Sardinian) and Wintermaker (Ojibwe).^16^ Nor are all constellations formed in patterns in the stars. ‘Dark constellations’, including Aboriginal Australian constellation Gawarrgay (The Emu in the Sky), and the Incan constellations Atoq (Fox) and Llamacñawin with Unallamacha (Eye of the Llama with Baby Llama),^17^ are found in The Great Rift (a series of dark patches in the Milky Way most visible in the southern sky).^18^ Neither, it seems, is the number of (unofficial) constellations fixed. In 2018, NASA sought to capitalise on the ongoing fascination with the final frontier, announcing the ‘discovery’^19^ of 21 ‘new’ constellations, including Godzilla, the Starship Enterprise and Schrödinger’s Cat.^20^

As we will delineate further in the next section, understanding these features of constellations can provide intellectual inspiration for an approach to feminist legal history that reveals new dimensions of feminist legal history. Here, we make two key points. First, crucially, a feminist constellations approach to legal history does not require drawing connections between feminists *per se* (although this might be the case, depending on the historian’s line of inquiry). Rather, it requires the legal historian to engage in the substantively and methodologically feminist *process* of drawing constellations: to draw not simply constellations of feminists, but *feminist* constellations—constellations that inevitably will, at times, encompass campaigns and reformers who were not feminist, and perhaps even explicitly anti-feminist. Second, as part of this process, we draw on the potential of identifying constellations beyond formal, internationally sanctioned patterns, that is, constellations like Buffalo Embryo and Wintermaker, which challenge dominant Western epistemologies and frameworks. We do so in recognition of the fact that, just as the Ancient Greek maps which have come to dominate (at least Western understandings of) the night sky can be dislodged, so too histories of women’s oppression can be rewritten, depending upon where and how the roots of such oppression are traced. Moreover, the process of seeking out dark constellations—that is, the spaces and shapes formed by the *absence* of stars—requires us to think in new ways about how to locate those rendered invisible by feminist scholarship and/or in legal history more generally. As such, the drawing of feminist constellations contributes to feminist demands to expose and challenge law and (historical) narratives of law as pro-imperialist, and/or as prioritising the activities of Western white women at the expense of others.^21^

### B. Asterisms

Asterisms are smaller networks of stars identified alongside, and sometimes within, the pattern of a constellation. These are distinct from the constellation within which they fall, though they may share one or more stars with it. Unlike the International Astronomical Union (IAU) constellations, asterisms are not strictly defined, with the result that there are many more asterisms than constellations.^22^ Asterisms also feature as part of ancient and indigenous constellations. The seven bright stars in Ursa Major which form an asterism known as the Plough in Europe, for example, which appears on the 13th-century Tomb of Seti I, are known as The Caribou or Reindeer in the Inuit tradition, the Rudder in Vietnam and Crustaceans in Myanmar.^23^

Asterisms serve as interpretive tools through which astronomers make sense of the sky, their form and spatial location depending upon the observer’s perspective. Asterisms do not seek to carve up the night sky into a series of agreed groupings, each with their own distinct and impermeable boundaries. So, their existence allows for the recognition and acceptance of an unlimited array of stories, myths and beliefs intersecting across the night sky, challenging the official demarcations across the centuries by cosmic cartographers. A feminist constellations approach to legal history embraces the inclusivity and flexibility of asterisms as a means of exposing the extent to which interpretations of law are themselves historically situated, shaped by the scholar’s standpoint. Put another way, as we shall see, a feminist constellations approach harnesses the language of asterisms, foregrounding a contextual and relational mode of understanding, as a means of making visible, and challenging, the interpretive processes through which dominant legal histories are constructed and constrained.

### C. Bright Stars

Astronomers use bright stars, or ‘supergiant stars’, which are visible with the naked eye, as a means of identifying a particular constellation typically through a process known as ‘star hopping’. This involves using the brighter stars (and the constellations or asterisms of which they are part) ‘as “jumping off” points to less obvious and fainter regions or objects of interest’.^24^ While some IAU constellations host one or two ‘supergiant stars’, most constellations do not.^25^ Nor is the brightest star within a constellation fixed. Rigel, for example, usually the brightest star in Orion, is sometimes overtaken by Betelgeuse. All constellations, however, have their own brightest star, or *lucida*. And, of course, while the brightest stars may easily attract our attention, many other stars are not visible to either the naked or the scientifically enhanced eye: on the average night in the UK, we are only able to see up to 1400 stars, a tiny percentage of the 200 billion trillion in the universe.^26^ This mode of understanding astronomy is equally instructive for feminist legal history. It invites us to use the figureheads or brightest stars within feminist constellations as tools for discerning the often overlooked, yet pivotal, contributions to law reform made by those whose traces within legal history remain faint, or unseen.

So much for talk of space and the tools and insights of astronomers. We recognise that legal historians might be forgiven for wondering what the formation of astronomical constellations has to do with legal history—let alone how constellations, feminist or otherwise, might improve our ‘doing’ of legal history. Our purpose here is not to rival Maggie Aderin-Pocock or Jean Luc Picard in their knowledge of the final frontier, nor is it to seek to persuade legal historians to abandon their discipline. Rather, it is to push gently at disciplinary constraints, and to provide the foundations for how we might begin to think about feminist legal history *as* constellations—what this might look like, and how feminist constellations differ from and/or draw on their celestial counterparts. As we leave discussions of astronomy behind, it is to this that we now turn.

## 3. Delineating Feminist Constellations

While astronomers use star atlases to map the constellations and patterns that organise and make sense of the night sky, there is no ‘star atlas’ of feminist campaigns that have shaped the law. However, common to both feminist and celestial constellations is a pattern imposed on the sky of stars which appear near to each other, but which often are not:^27^

The groups of stars that made up the … constellation patterns have, in most cases, no physical connection with each other. All constellations are a matter of perspective. They are simply our Earth-based interpretation of two-dimensional star patterns on the celestial sphere, composed of stars of various different brightnesses and at widely differing distances from Earth.^28^

All constellations and asterisms—feminist or otherwise—are then arbitrary, constructed and culturally specific two-dimensional patterns formed either by bright and invisible stars or, in the case of dark constellations, in the negative space created by, and existing alongside and intersecting with, those drawn by in the starlight. They are earthly interpretations imposed on the sky (or, in the case of feminist constellations, on history) to aid *our* understanding, to give meaning to, or to facilitate, *our* specific endeavours; a means of imposing some form of orderly relationship on something much more complex. Just like their celestial sisters, feminist constellations are dependent on *our* positioning, upon *our* location *now*, on who or what we take as our centre or jumping off point, their topics or themes informed by the priorities and needs of those below. Like their other-worldly equivalents, they provide a means of navigating ways forward, of finding new purposes and anchors. And, like the mixture of animals, mythical characters and inanimate objects that circle above our heads, feminist constellations might form around more or less anything: a common endeavour, person, location, time of day, organisation, employment, novel, statute, case, protest and so on.^29^ At other times, we might explore feminist asterisms within larger constellations, for example, the role of the ‘No More Page 3’ campaign within the broader feminist fights against pornography.^30^

Feminist constellations are inclusive. There are no criteria for inclusion (or exclusion)—beyond a common interest in the given theme. A feminist constellation is not simply a network of allies or comrades (unless that is the specific intention), but rather of insiders and outsiders, of rival groups and opposing views. The position (or exclusion) of a particular individual, organisation or event within a given constellation might well be fraught, contentious and uncomfortable: ‘Enemy feminists^31^ are positioned alongside ‘heroines’, and ‘heroines’ within multiple and, at times, troubling constellations. Feminist constellations are inevitably (and rightly) contested. They are fragmented and incomplete; their formation limited by our own knowledge and interests, our own positioning. They are patterns drawn—and connections made—in the knowledge that for every person, protest and event for which there are records—there are many others that have been ‘lost’ or silenced.

Nor are feminist constellations concerned only with feminists and/or feminist activism. It is not that the components of the constellation are feminist, but rather the *approach* is feminist in that it brings gender and power into history’s mainstream. A feminist constellation of Caxton Hall, located on the corner of Caxton Street and Palmer Street in Westminster, London, for example, might include members, supporters and opponents of the Women’s Social and Political Union (WSPU), the Homosexual Law Reform Society, Indian independence and the National Front.^32^

Moreover, unlike their IAU-approved counterparts, feminist constellations are not fixed. There is no agreed or exhaustive list of feminist constellations. Their boundaries are neither prescribed nor restricted. Rather, feminist constellations mirror the mix of official, indigenous, light and dark, ‘new’ and ancient constellations swirling above our heads. They are messy, fluid and overlapping. There is no requirement that each star (which might be an individual, event, endeavour, campaign, location, case, statute and so on) belong to just one—or, indeed, any—constellation.^33^ Nor must feminist constellations stay within their celestial boundaries. Rather, they can spill out across the sky, intersecting with others as they draw their patterns in the sky: a butterfly, perhaps, for the Latin American feminist movement’s march against gender-based violence,^34^ a teddy bear for the women of Greenham Common,^35^ a spider for Lady Hale^36^ or a Ford Cortina for equal pay.^37^

A feminist constellations approach encourages us to look beyond the familiar and taken-for-granted, to explore and make visible dis/connections across space and time. The drawing of constellations requires the legal historian to actively seek out connection between and beyond individuals, organisations and groups as a means of articulating new narratives, plotting new ways forward. With this in mind, we turn in the next section to how we might begin to map feminist constellations in law.

## 4. A Feminist Constellations Approach to Legal History

The language of constellations, of how astronomers have mapped the galaxies around us, provides us with a novel and effective way of *doing* legal history. It is not (simply) a specialist exercise in doctrinal ‘excavation’.^38^ Nor is it (simply) about ‘finding’ the women in stories of law reform missing in traditional legal histories, collecting and displaying them as a lepidopterist might do their butterflies. Rather, a feminist constellations approach pushes at the boundaries of legal history. It extends understandings of ‘legal’, ‘law reform’ and ‘success’, subverting institutional narratives to include women’s activities outside the courtroom and Parliament—those taking place in front rooms, on factory floors, in domestic violence shelters, in the streets or on the silos of Greenham Common. In so doing, it provokes a different mode of thinking about law reform altogether, challenging the notion that law making and reform are limited to specific ‘agents’ such as legislators and judges, so that we can see legal change as something participated in by everyone.^39^ A feminist constellations approach to legal history embraces and utilises the disorderly, confronts the uncomfortable and makes space for the unknowable, requiring us to explore, in Elizabeth Grosz’s words, ‘what could be but does not yet exist’.^40^ To this end, a feminist constellations approach to legal history has five interlinked characteristics: (i) the recognition and embrace of multiple temporalities; (ii) the making visible of neglected continuities; (iii) the amplification of inconspicuous success and impact; (iv) a willingness to engage in the process of star hopping; and finally (v) the use of constellations as a system of navigation.

It may be that one or more of these characteristics is familiar. Each of these elements shares some similarities with established historical approaches. Temporality, for example, has long been recognised as a gendered construct,^41^ and over the years many historians have attempted to rethink periodisation in novel ways.^42^ Similarly, the suggestion that neglected continuities might be made ‘visible’ accords with those who pursue interconnected histories, which can rethink interactions between feminisms and broader global politics,^43^ while the process of engaging in star hopping, that is, of using bright stars or figureheads to find less visible activists, can be compared to a micro-history approach, which uses a small-scale subject such as an individual or event to understand how larger cultural or social forces have had impact.^44^ This is not surprising. Nor is it particularly problematic. It would be a strange *feminist* approach to legal history to suggest or present our insights as entirely novel. After all, feminism and feminists are forged in and by the battles and arguments of those who have gone before. Take, for example, the 1970s *Spare Rib* editorial collective, who, as Julie Sauvage explains, deployed history as a shared narrative through which feminist generations might ‘relate to each other by making and sharing a common time that is both lived and narrated. History thus becomes a poly-temporal, collective experience, in which generations can be conceived as overlapping spaces, rather than successive eras’.^45^ Feminism—and, in turn, feminist method and history—is an explicit and deliberately cumulative and collaborative endeavour. So too is our feminist constellations approach to legal history. However, none of the approaches referenced above have sought to combine the five elements into a coherent and robust methodology, nor have they been developed as part of *legal* history, as a means of reassessing and deconstructing the more standard accounts of law reform. It is on this basis that we outline the substantive and methodological insights of our approach below.

### A. Embracing Multiple Temporalities

Exploring feminist networks across time contrasts with—and resists—the temporality imposed upon feminist campaigners and the women’s movement by historically limiting descriptions such as ‘First Wave’ and ‘Second Wave’. As Joanne Conaghan notes:

The ‘periodization’ of feminist thought and activities is undoubtedly a tidy way to order and present development and change within feminism over a broad time frame. However, the use of such temporal indicators to situate theoretical and conceptual positions is not without problems. In particular, it can lead to the neglect of evidence suggestive of a contrary or more complex picture of the period under scrutiny. Periodization functions as a form of teleology, discouraging exploration of historical complexity and downplaying the presence of tensions and contradictions in historical accounts. Periodization also fosters a tendency to contrast and oppose as a way of constructing boundaries. For periodization to be meaningful there must be a beginning and an end to a period, followed by the beginning of something new again. Periodization demands juxtaposition.^46^

The result, Conaghan continues, is ‘to yield random and potentially misleading temporal demarcations through which historical events are subsequently interpreted’.^47^ In resisting and resetting temporal limits imposed by traditional accounts, a feminist constellations approach to legal history sits alongside the growing body of academic literature in exploring the limitations and possibilities of legal temporalities.^48^ It recognises time and existing temporalities as ‘discontinuous and fragmented’.^49^ And it accepts, with Emily Grabham, that time is neither ‘“natural” nor “social” but always both, brewed through changing relationships of humans and material forms’.^50^ It recognises temporalities as ‘complex and multiple’ entities, through which the historian can situate ‘women’s relations with the world in which they campaigned, learned, lived and worked’.^51^

None of this is to suggest that time is unimportant.^52^ Nor does it advocate for an approach to history that is out of time, that ignores or downplays the importance *when*, for example, a particular organisation was operating. Far from it. Rather, a feminist constellations approach adopts an understanding that resists linear approaches, where waves, periods, ages and such like come one after the other, and where key events and people are ‘frozen’ at a particular time and place, and that recognises time as complex and contested but nonetheless (or maybe even as a result of this) essential to our understanding of history.^53^ It seeks instead to deploy the flexibility provided by imposed celestial frameworks to draw new constellations in legal history’s narratives, to identify multiple, overlapping constellations in which individuals and groups, organisations and networks are located at various points across timelines, and beyond tempos and rhythms.

A feminist constellations approach can make visible, for example, the relationship between women’s challenges to the Norman ‘forest law’ introduced by William I and the Women’s Peace Camp at Greenham Common as forms of ongoing civil resistance by women to ensure physical security, health and wellbeing of their families. In making links between ‘mainstream’, well known, institutional aspects of legal history, a constellations approach can demonstrate how apparently disparate events are inextricably linked to feminist campaigns across time and, as a result, to the gendered power dynamics at the heart of law. It reveals the ongoing impact of ‘past’ campaigns, such as that of the all-woman Society for the Protection of Birds, led by the anti-feminist and vehemently anti-suffrage Etta Lemon, and Emmeline Pankhurst’s leadership of the WSPU, on current campaigns to protect wildlife in the face of climate change.^54^ It connects contemporary campaigns seeking to secure women’s access to public spaces, placing the global Reclaim the Night marches^55^ alongside the Mumbai-based Why Loiter? campaign,^56^ the Girls at Dhabas in Pakistan^57^ and Women in Urbanism Aotearoa initiatives.^58^ It also shines a light on the connections between the Peasants Revolt of 1381, started not by Wat Tyler but by Joan Hampcok and Agnes Jekyn over the imposition of tax on married women,^59^ and the campaigns on the same issue by the Married Women’s Association (MWA)^60^ and the Women’s Budget Group, founded by Georgina Ashworth almost seven centuries later.^61^

A feminist constellations approach, like much of feminist legal history, disrupts Whiggish tales of progress and replaces Dickensian portrayals of heroes and heroines,^62^ of feminist success and recognition in the face of adversity, with ones that are messier, more complex but no less—and perhaps even more—inspiring. It emphasises the *un*timeliness of many feminist campaigns, allowing us to reset the clock—to shift our starting points or, better, to see time in flux.^63^ It provides us with a way of resisting the imposition of time (or, more often, the absence of time) on us in suggestions that those agitating for change and/or recognition might need to be patient. And, in drawing attention to the extent to which ‘progress’ ebbs and flows, it makes plain the extent to which ‘new’ rights are in fact the restoration of ancient rights: whether it be the property rights of women pre-coverture,^64^ the extension of the franchise,^65^ the ability to sit as judges or jurors,^66^ or equal pay.^67^

This is important. Conventional legal history might treat certain reforms as temporally and, therefore, logically interconnected. The campaigns to reform married women’s property rights from the 1850s through to the late 19th century offer a familiar example: their chronological proximity often leads us to presume a coherent reform trajectory.^68^ Yet this assumption reflects dominant narratives. By contrast, a feminist constellations approach resists confining analysis to such linear temporal frames. Rather than drawing temporal lines only within established themes (such as the ability of women to own property or to participate in civic society), a constellations approach reconceives temporality itself. It allows for connections that unfold across much longer arcs, as well as those that surface between seemingly disparate developments occurring in close succession.^69^ A feminist constellations approach can therefore reveal structures that constrain efforts and prevent change.^70^ It demonstrates that different battles are fought on the same ground, and are lost and won, again and again and again. It takes a long view, enabling us to see how and when feminist efforts to reform the law were more likely to be successful. It shows how, for example, legal pragmatism has been employed by feminists over time, as well as the conservative and increasingly neoliberal forces constraining feminist efforts to reform the law today—laying bare new possibilities for overcoming the impasse feminists have faced historically when attempting to reform the law.

### B. Disclosing Neglected Continuities

To look at the night sky is to look into the past and at the present, and to imagine the future. It is to look back in time, at the constellations, and the stars that form them, not as they exist now, but as they did then. It is to travel back in and across time. This provides an apt metaphor for connections between feminist groups, so that they are understood not just within the time they campaigned, but also within the bigger history of the women’s movement both then and now. Vitally, in looking for articulating feminist connections and continuity (which may also be messy, *ad hoc* and fragmented) across themes, concepts, organisations and campaigns, we can highlight feminist legacies in ideas that are often *considered* to be ‘new’ but are in fact not. Put another way, if embracing multiple temporalities allows us to see the ‘bigger picture’—connected disparate and often starkly contrasting groups across centuries—exploring neglected continuities is about exploring the detail, and the smaller, more specific connections across time periods and between groups and campaigns.^71^

One example of this is the continuity between the ‘wages for wives’ campaigns in the 1930s and 1940s and those that have been calling for ‘wages for housework’ since the 1970s. At the height of second-wave feminism, the International Feminist Collective (IFC) began to call for the recognition of different forms of labour performed by women—including ‘wages for housework’. A key motivation was the ability of women to make visible their role in capitalism by demanding payment for work historically done for free, which in turn would destroy the distinction between work in the marketplace and in the home.^72^ Yet few, if any, members of the ‘wages for housework’ campaign acknowledged—or sought to explore—connections with earlier groups or organisations, to draw support from feminist endeavours across time. Indeed, Louise Toupin, a member of the Quebec Women’s Liberation Front, even went so far as to declare ‘the Wages for Housework perspective’ to be ‘a completely original school of thought, and a toolbox for action, at the beginning of second-wave feminism’.^73^

It was, of course, anything but. To suggest that the 1970s feminist movement had happened upon something entirely novel is simply incorrect. The MWA—a British group established in 1938, adopted the slogan ‘wages for wives’ in its early years.^74^ And even then, arguments that economic independence was essential to women’s position as citizens were long-standing. Charlotte Perkins Gilman, for example, had argued in 1898 that ‘wives, as earners through domestic service, are entitled to the wages of cooks, housemaids, nursemaids, seamstresses, or housekeepers’.^75^ The phrase ‘wages for wives’ had been used in an argument for a statutory ‘living wage’ for wives in 1907,^76^ and again in a newspaper report in the same year suggesting ‘The woman who has no money of her own and no means of earning any’ was ‘a slave’.^77^ It was also the subtitle and subject matter of a play by Guy Bolton, which had been turned into a popular ‘motion picture’ in the mid-1920s,^78^ and had been argued for by Eleanor Rathbone MP at the National Union of Societies for Equal Citizenship summer school in 1928.^79^

By mapping constellation patterns whereby feminists have demanded a wage for domestic labour across space, place and time, we might better recognise the evolution and disunity of similar ideas and campaigns. We are better able to make links between different campaigns illuminating commonalities, for example in terms of the social conditions which made the issue at hand to be a pressing social issue *at that particular time*. We can recognise ideological differences between campaigns: juxtaposing the MWA’s call for the support of wives by their *husbands* with the Marxist-feminist-inspired IFC demand that wages for housework be paid by the *state*.^80^ We are also able to locate these campaigns as asterisms within a broader long-standing constellation concerning the poverty of women, particularly in heterosexual relationships where they are dependent on men, and the need for women to have money of their own to be liberated (a truth recognised by all feminists—including, most famously perhaps, Virginia Woolf).^81^ Finally, we can make concrete connections between these historic campaigns and current feminist and social justice arguments, relating to the redistribution, recognition and valuing of unpaid care in the home and broader community,^82^ including, for example, a scheme in 2021 analogous to wages for housework that was promised to 30 million women in several Indian states, in the form of cash transfers that implicitly recognised their work in the home.^83^

Once again, this is important. Failing to see the continuity, however slight or conflicted, between the various groups, arguments and campaigns—for example, by presenting each campaign as a fresh start or new idea—only downplays or ignores the achievements of those who have gone before, and not only presents an inaccurate and misleading history of feminist reform, but vitally actively *benefits* deeply embedded political and societal institutions and structures. It ensures that each campaign starts from zero, allowing those resisting more fundamental or immediate change to maintain the status quo for longer. It denies feminist activists and scholars the strength that is gained from knowing and using their roots, the ability to place themselves on the shoulders of those who have gone before. It weakens their arguments, and makes securing change harder.

### C. Amplifying Inconspicuous Success and Impact^84^

In his discussion of women under the English law published in 1896, AR Cleveland suggested ‘it is very questionable whether woman has ever gained any great concessions by direct agitation’.^85^ He continued, ‘If we look back, and note the numerous changes in the laws concerning women, how many of these changes are attributable to women themselves?’^86^ Even recognising with Rackley and Auchmuty that the best way to ‘remove the threat of feminist agitation and deter future attempts by women to secure legal change by their own efforts is to deny previous successes’,^87^ Cleveland’s understanding of law and law reform is spectacularly myopic. He might be forgiven, perhaps, for failing to predict the future importance of the strikes by match women and girls at the Bryant & May match factory on later campaigns for equal pay,^88^ or even (though this is more of a stretch) for thinking that the ridicule and disdain that greeted the first suffrage petition, presented by Henry Hunt MP in August 1832 on behalf of Mary Smith, might persist.^89^ But we should be less charitable about his failure to recognise the law reform success of his direct contemporaries. Campaigners such as Elizabeth Fry and other members of the ‘Ladies Association for the Improvement of the Female Prisoners at Newgate’, for example, whose insights relating to the treatment of women prisoners are directly reflected in the content of the Gaol Act 1823.^90^ Or the impact of Barbara Leigh Smith Bodichon, Caroline Norton and Frances Power Cobbe’s long-running campaigns seeking legislative reform of marriage laws on both the Divorce and Matrimonial Causes Act 1857 and Married Women’s Property Act 1882. (Both Acts are mentioned in Cleveland’s book; Bodichon, Norton and Power Cobbe are not.)

Of course, Cleveland is not alone in overlooking the contributions of feminist and women’s groups and organisations.^91^ As Felice Batlan notes:

It was long acceptable to write legal history, even excellent legal history, without including women or gender. Legal historians rationalized that because women did not participate in the ostensibly most significant events of legal history … they were irrelevant when writing ‘serious’ legal history. While women might play a role in a social history of the law or in discussions of domestic relations law, on the whole, women and gender stood at the periphery of legal history.^92^

This is not the case today. However, while feminist and women’s engagement with law and law reform is far more visible than it once was, the ‘inconspicuous impact’ of feminist and women’s campaigns and groups remains less so.^93^ Impact that, in Sharon Thompson’s words, ‘thinks the unthinkable’, that is, untimely, non-linear, arising from failure and often piecemeal.^94^ Impact, too, that may not have been successful at the time—often not for want of trying by the women involved—but that nonetheless contributed to change that happened later.

A feminist constellations approach requires us to search for—and amplify—the ways in which feminist pressure groups’ engagement with law and law reform has consciously or otherwise been rendered inconspicuous. It requires us to look in unusual places, to hunt out activities and efforts that went unseen, that were unremarkable and were often unsuccessful. It adopts, with Krista Cowman, ‘a much less confident but more nuanced picture of multiple progressions and successes interspersed with periods of stagnation, defeat and even regression’.^95^ In seeking out connections across time and space, it allows for a broader, more contextual understanding of legal change; one that recognises, with Lena Jeger MP, that while

[m]uch of history is a story of elusive, intangible shifts in mores, in changes in public habits and opinions and attitudes which defy analysis … It may be that the successful campaign is successful only when it coincides with the imperceptible tide. But are the imperceptible processes of themselves ever successful in the practical course of progress? Can they ever manage without the campaign? I think not.^96^

In amplifying previously inconspicuous successes and the impact of feminist/women’s organisations and campaigns, a feminist constellations approach takes a long view of feminist success and endeavours. It stands against the complacency of legal/historical accounts presenting women as passive recipients of social change and reform rather than instigators of it. We might explore, for example, how the sustained feminist critique of multiculturalism articulated by Southall Black Sisters, an anti-racist, feminist advocacy and campaigning organisation since the mid-1980s, led to the recognition of forced marriage as a specific form of violence against women, and a shift in government policy away from criminalisation in 2007.^97^ Or how feminist campaigners and academics came together in the early 2000s in response to the government’s plans to criminalise the possession of what it termed ‘extreme pornography’ to achieve a legal change that made it illegal to possess pornographic images of rape over a decade later.^98^

More than this, a feminist constellations approach to legal history challenges what we recognise as success. It requires us to look in different places, to go beyond representing feminist activities—successful or otherwise—as isolated endeavours and to reframe earlier ‘failures’ as part of later success—or even further failure.^99^ It also requires us to see earlier campaigns as part of a longer-term endeavour and contributing—in many cases—to a *better* understanding of the law reform processes. And it allows for the recognition of campaigns that were not successful at the time, but which are nevertheless part of the movement towards a particular aim. Examples include the failed implementation of the recommendations in the1919 Report of the War Cabinet Committee on Women in Industry, relating to the wages and employment conditions of women, and Ethel Short’s legal challenge to the marriage bar that prevented married teachers from working in schools (reported in *Short v Poole Corporation* (1926)), both of which played a key role in demands for equality of pay and opportunity in the workplace.^100^

Of course, the slow, often *ad hoc* and serendipitous progress (or lack thereof) of feminist campaigning is not new. Elizabeth Wolstenholme Elmy spoke out against rape in marriage over a century before it was criminalised in the UK.^101^ The first formal national organised movement for ‘Votes for Women’ began with a mass petition in 1866—some 62 years before the Equal Franchise Act.^102^ The Married Women’s Property Act 1964 was the culmination of a 20-year campaign led by the MWA to ensure that money derived from a housekeeping allowance (given to the wife by her husband) and property bought with that money belongs to them *both* in equal shares.^103^ The Sexual Offences Act 2003 was the culmination of over 30 years of campaigning by feminist activists, scholars and organisations for rape law reform.^104^ However, in placing feminist endeavours—successful or otherwise—across time and within the ebb and flow of past, present and future law reform, a feminist constellations approach allows us to see the transformative possibilities of feminist strategies to change the law. This is important, not least because, as EP Thompson notes, ‘in some of the lost causes … we may discover insights into social evils which we have yet to cure’.^105^ A feminist constellations approach opens new possibilities for what we mean by justice and reform, and how it might be achieved. It takes seriously the view that law and law reform are not something done for women, by others. It challenges assumptions about how legal change comes about, and the role of ‘ordinary’ women and men in this, highlighting the importance of grass-roots pressure for, and at times resistance to, change. It resituates ‘law’ not as something external, something to follow, break, rebel against or argue about, something that is made within institutions and by institutional actors, ‘originating outside of [our] lives and exerting influence on them’,^106^ but rather something through which women and feminists might do what Máiréad Enright, Kathryn McNeilly and Fiona de Londras have described as ‘feminist law work’.^107^

### D. Star Hopping Within and Across Constellations

Despite current apathetic trends towards feminist heroines and role models by some feminist legal historians, feminists have not always had a problem with heroines. Consider, for example, Cicely Hamilton’s suffrage play, *A Pageant of Great Women*, which featured Hypatia, Jane Austen, Grace Darling, Joan of Arc and Boadicea, among others.^108^ Nevertheless, it is clear that for a while now, relations have been decidedly frosty. It is time, we are told, to let go of the tales of ‘exceptional’ or ‘rebellious’ women in which the heroine defeats the odds and slays the patriarchal dragon.^109^ We should move on from tales in which flaws are airbrushed away, where darker undercurrents lurk, at best, uncomfortably in the margins and, at worst, disappear from view, and in which the advantages of, for example, family, money, ethnicity and the collective efforts of others go unacknowledged. And quite right too. As the women of the Greenham Common Peace Camp knew, when we focus *only* on the bright stars—whether for censure or praise—we fail to see most of the stars in the sky.^110^ After all, only a fraction of stars in the sky are visible to the naked eye, even fewer make up the pattern of a constellation and some—most obviously the dark constellations—have no stars at all. So too in legal history. In focusing only upon the thoroughly documented lives in law, as all good (feminist) legal historians know, the stories of women and feminists on the fringes of these accounts are at risk of being buried. Mapping the constellation of relationships, networks, connections and so on within which that individual worked and lived has been an important feature of (feminist) legal history and biography for some time.^111^

A feminist constellations approach requires legal historians to engage in the process of ‘star hopping’. It starts from the position that the trouble with feminist heroines is not so much their existence, but what we do with them. Rather than looking directly at them and allowing ourselves to be dazzled by their brightness, we should use their light, their brightness, to find others, for example by mining the archives of prominent feminists to learn about their connections and relationships with others. As such, a feminist constellations approach to both legal heroines and the researching and writing of legal biography differs in emphasis from that followed to date. Rather than placing an individual (or organisation, campaign and so on) at the *centre* of the work and mapping their networks and connections out from there—their inclusion simply a by-product of telling their story well—a feminist constellations approach uses the individual heroine (or star) as simply a jumping-off point—a lens through which we might find others, and discover more *about the constellations* of which they were part. This not only shines a light onto organisations and individuals hidden in the shadows, but also allows us to see those cast as role models and icons as complex individuals—often with uncomfortable, darker sides—and sitting in multiple (and at times conflicting) constellations. After all, as Katie Pickles points out in her exploration of heroines in history, those who become icons or stars are not necessary role models.^112^

The work of Sundari Anitha, Ruth Pearson and Linda McDowell exploring the legacy of Mrs (Jayaben) Desai and the Grunwick workers’ strike is an example of how we might engage in the process of star hopping. Jayaben Desai was the instigator, coordinator and leader of the Grunwick workers’ strike in 1976. In 2016, she was named one of seven most influential women of the last 70 years by the BBC’s *Woman’s Hour*.^113^ Desai, a Gujarati woman from East Africa, became the face of a two-year struggle that made visible not only the poor working conditions of her predominantly South Asian female colleagues working in the Grunwick film-processing factory in north west London, but also the sexist and racist discrimination they endured.^114^ Described by one activist as ‘the Ascot of the Left’, somewhere where it was ‘essential to be seen’,^115^ the Grunwick strike now has iconic status in the history of the labour movement of migrant workers and labour law more broadly.^116^

As the life history interviews with now elderly Grunwick strikers conducted by Anitha, Pearson and McDowell reveal, Desai’s star illuminates a constellation of relationships and networks both within and beyond the Grunwick factory. These go beyond treating the Grunwick strike as simply ‘an emblematic turning point’ in the representation of black and minority ethnic women workers, as an event confined to that moment in time (though it was and this is important), and reveal far more than Jayaben Desai’s story and the incident of the strike itself. By following the light of Desai’s star, we not only ‘find’ others within a particular constellation—Parminder Kaur, Mohinder Kaur, Hanso Kaur and Guro Kaur, for example, who were elected shop stewards following a 10-week strike in 1982 by mainly South Asian women at Supreme Quilting in Smethwick in the West Midlands^117^—but also, in focusing on the multiple constellations within which Mrs Desai and the Grunwick strike might sit, we are better able to develop more holistic understandings of, for example, histories of migration and immigration, and (anti-)racism within the trade union movement, as well as the impact and ongoing legacy of women strikers in effecting social and legal change.^118^

As well as illuminating ‘new’ stars within new and existing constellations, the process of star hopping also offers a solution to a familiar ‘problem’ or challenge of doing women’s history: the absence of women in the archives—or at least those whose lives were not important enough to preserve, who were not powerful and distinguished, who were not queens, firsts or otherwise exceptional. A feminist constellations approach requires feminist legal historians to be creative. Rather than treating the women as ‘missing’ (a framing that at best downplays the institutional hierarchy and exclusion at play and at worst suggests some level of culpability on the women themselves for not being sufficiently notable) or as presenting an insurmountable obstacle to recovering their stories, it requires them to look elsewhere, to reorientate their focus, in order to uncover ‘hidden’ women and to understand their significance. They might look, for example, to letters of support, such as those sent by Mrs Gould and S Helen Wigg-Gilbert to Nancy Astor following her election as an MP, kept by Astor (or someone close to her) and which ultimately made their way into her archive,^119^ or to court documents, such as the petitions submitted to the Lancashire quarter sessions courts detailing the efforts of Alice Brewer and other mothers in caring for non-kin children in the north of England,^120^ or those submitted to the Court for Divorce and Matrimonial Causes detailing the reasons why women were seeking divorce.^121^ The result is that a feminist constellations approach not only makes the previously hidden women visible, adding their ‘star’ to a given constellation/s, but also deepens our understandings of broader issues and concerns, including, to use the examples above, attitudes to women’s representation, legacies of care, social and economic hardship, and gendered violence.

#### (i) Dark constellations

The process of star hopping across and within constellations also creates space to recognise the contribution of women yet, or never, to be ‘found’. It allows us to ‘see’ the predominately working-class women, rural women, illiterate sisters, mothers, spinsters and children, who have not left behind swathes of rich source material in the mass of stars extending across our galaxy as well as in dark constellations, formed in the negative space created by, and existing alongside and intersecting with, their more familiar counterparts drawn in the starlight. Dark constellations are not dependent on the visibility or identification of particular stars. They are visible because of the general light from all the stars in the sky. To see them, we need to shift our focus away from looking at—or for—stars and towards the darker patches of the sky formed by clouds of gas and which hide the stars within them. It is here that we find dark feminist constellations intertwining with, within and alongside their glistening counterparts, collectives of unknown women on whose shoulders we stand; women who came together for a specific purpose—women who, for example, marched from Cardiff to Greenham Common in August 1981, who participated in Kvennafrídagurinn (Iceland’s Women’s Strike) in 1975 or the Green Belt Movement in Kenya, who wrote letters that have not been kept and/or who have volunteered, and continue to volunteer, at Rape Crisis, Women’s Aid, Pro Mujer, the Pratthanadee Foundation, Planned Parenthood and other frontline charities and organisations in the UK and worldwide.

At the same time, the presence and identification of dark constellations challenges us to shift our focus away from dominant feminist (historical) narratives and to acknowledge the extent to which historical narratives used by white feminists are tied to imperial histories rooted in the prejudices of colonial periods. It requires us to sit with, and then to challenge, the extent to which, in Valerie Amos and Pratibha Parmar’s words, such narratives suffer from ‘the same form of historical amnesia of white male historians’ because they ignore ‘the fundamental ways in which white women have benefited from the oppression of Black people’.^122^ Dominant histories of the woman’s peace camp at Greenham Common are, Amos and Parmar suggest, a case in point. Their narratives, focusing upon the women’s peace movement and the Campaign for Nuclear Disarmament, unwittingly or by design downplay or outright ignore the extent to which both were tied to nationalist sentiments (‘we want to protect our country’), obscuring the political priorities of Black women as well as international issues such as Britain’s illegal mining of uranium in Namibia. Not only are such narratives partial, they are also distorting: Black women within the Greenham Common peace campaign are marginalised into ‘the role of caterers’, in contrast to the dominant presentation of the Greenham Common women as activists or agitators.^123^

The process of star hopping and exploring the dark constellations actively pushes us to pursue this approach in our work, to look away from and behind prevailing narratives, to define feminist legal history in new and different ways, and to ‘challenge the use of some of the central categories and assumptions of recent mainstream feminist thought’.^124^ After all, when we look to the sky, it is not the few that form the pictures of the constellations or even the brightest stars that first get our attention or capture our imagination, or even the 1400 or so that we can see with our naked eye, it is the sheer magnitude of the 200 billion trillion visible and invisible stars across the universe that takes our breath away.

### E. Navigating the Stars

Of course, constellations—feminist or otherwise—are not simply pictures in the sky. One of the most familiar ways the constellations are used is as a system of navigation.^125^ For centuries, adventurers, colonialists, merchant sailors and traders, birds and other animals have followed the stars to navigate their way around the globe, to journey to far-off places and find their way back home. Today, sextants are used alongside satellites by governments and the military to protect us in the event of a cyber-attack.^126^ And at the University of Victoria in Canada, there is a ‘sky classroom’, featuring a large skylight that frames the night stars and literally turns to the night sky as a site of legal learning. In the classroom, students explore how Indigenous legal principles are derived from constellation patterns and the stories they encode, so they can consider how such frameworks might inform and reshape Canadian common law.^127^

Similarly, through engaging with Indigenous and traditional star atlases in legal thinking to identify and explore feminist constellations, dominant legal narratives and conceptions of justice are challenged.^128^ This provides us with new pathways for reimagining law and advancing legal reform, as well as a means of navigating past, present and future issues relating to social justice, feminism and change. Its purpose is both illustrative—demonstrating familiar and unfamiliar links and connections between the individuals, organisations and events that were, perhaps, not previously visible—and purposive—requiring us, like the travellers of old, to *do* something with the constellations we draw. Just as the travellers needed to understand what they were looking at to be able to use them, so do we.

Feminists have long understood their place in history, and have used history to guide their efforts to change the future, to better understand the material forces constraining their own efforts and as a means of legitimising or amplifying their own endeavours.^129^ As Krista Cowman notes, ‘Writing history became a form of activism’.^130^ Feminist historians, she continues, adapting Marx, have infused history with politics, simultaneously seeking to ‘interpret the world while trying to change it’.^131^ Take, to use Cowman’s examples, Christine de Pizan’s *Livre de la cite des dames* (c 1405) efforts to ‘counter medieval male supremacy by describing an allegorical female world where Queen Esther, Minerva and others combined to install a sense of possibility in women readers’, or Virginia Woolf’s urging of ‘female undergraduates to collect “a mass of information” to reconstruct “the life of the average Elizabethan woman”’ in response to the absence of women in GM Trevelyan’s new *History of England*, published in 1927.^132^ We see this too in the popularisation of history—and in particular the early 20th-century suffrage campaigns—in Women’s Liberation Movement (WLM) magazines and books.^133^ Take *Spare Rib*, the longest-running and most widely circulated of these magazines, which, for example, used history as a way of

creat[ing] a common, shared, feminist memory for its readers, and [to] … reinforce a sense of continuity and community … a generational space where the kinship between the feminists of the WLM and the suffrage movement no longer rested on lineage and succession, but belonged to a non-linear temporality and relied on affinity, shared experiences and mutual listening, which might be, at bottom, the very definition of *sisterhood*.^134^

We can see contemporary feminists doing the same. Suffrage iconography has been adopted by or referenced in association with a wide range of feminist endeavours.^135^ Newspapers have identified ‘new suffragettes’— for example, Awezan Nuri, a Kurdish poet fighting against female genital mutilation; the Chinese gender rights activist Haiyan Ye;^136^ and the Ukrainian women’s rights movement Femen.^137^ Suffrage motifs, statutes, WSPU colours and/or personalities have been adopted by present-day feminist campaigns, most obviously the Fawcett Society,^138^ but also Women Against State Pension Inequality (WASPI), based in the UK.^139^ Gender-critical feminists in the UK and Ireland have explicitly and deliberately presented their cause as something ‘the suffragettes would have wanted’^140^ or that ‘the Countess’ would not have fought for (referring to Constance Markievicz, the first woman elected to the UK House of Commons)^141^ in their efforts to appeal, or lay claim, to essential truths of equality, womanhood, nationalism and so on. As Sarah Pendersen notes:

Gender-critical feminists … make frequent references to the suffragettes and suffragists in their descriptions of their own situation … they … performatively invoke their heritage as militant, radical feminists, and imbue their cause with a legitimacy and heroism some contemporary feminists and the media would refuse them. By positioning themselves as the suffragettes, they are able to position their detractors as anti-suffragists (on the wrong side of history).^142^

A feminist constellations approach provides a counter to this. It challenges simplistic understandings of feminist history by (some) present-day campaigns, exposing partial or self-interested interpretations of past role models or campaigns, in which the past is framed ‘in terms of present-day needs and values’.^143^ But more than this, a feminist constellations approach to legal history reminds feminist (legal) historians of the political roots of their discipline. It holds feminist legal historians to account. To adapt Sandra Berns and Paula Baron’s formulation, it requires us to research and write in a way that cannot be bracketed; to speak neither as a (feminist) legal historian nor simply as a feminist (legal historian), but rather in a way in which prefix and noun intertwine with and inform the other.^144^ It holds us to our feminist *and* scholarly roots. It dares us not only to think more boldly about who makes, influences and reforms the law, but our role within this—our own place, and that of our research, in ‘carrying on the long tradition’.^145^ It requires us to take seriously the transformative obligations of feminist legal history and the extent to which this informs not only what and how we research, but what we do with it: where we publish, who we work alongside, the campaigns and causes we might amplify.^146^ In drawing attention to ‘feminist law work’ in all its forms, feminist legal history can enable law, its formal processes and what it means to ‘do law’ to be reimagined.^147^ It actively resists a ‘retreat’ to the academy. Instead, a feminist constellations approach locates legal history at the fulcrum of feminist activism—a bridge between the past, present and future, and a mechanism through which to secure social justice and change—and challenges feminist legal historians to cross it.

## 5. Conclusion: A Feminist Star Atlas

Speaking in 2024, Helen Sharman, the first British woman to go to space, talked about how doing so had shifted her perspectives. It had made her think differently about the things that were important to her.^148^ The same might be argued for adopting a feminist constellations approach to legal history. It forces us to shift our perspectives, to alter our priorities, to think differently.

When we look to the stars in the sky, the constellations we see depend upon our surroundings, our location and where we decide to look, and are all informed and shaped by our own positionality, bias and interests. Astronomers use star atlases, mapping constellations and patterns that organise and make sense of the night sky; others use fable and myth.^149^ Feminist legal historians have only their curiosity and imaginations, shaped by their political commitment to social justice and informed by their sources. There is no ‘star atlas’ of feminist campaigns that have shaped the law. Feminist constellations, like many of the campaigns and organisations they invoke, are far more *ad hoc*, criss-crossing the sky, spanning jurisdictions and temporalities, intersecting with and pulling against each other. As such, our feminist constellations approach faces the same challenge as that levelled at Sanjay Subrahmanyam’s connected history: how ‘to retain loving attention to specificities without losing grasp of the bigger picture, neither getting bogged down in particularities nor succumbing to the ease of a catch-all narrative that ignores the exquisite detail’.^150^ But this should not discourage us from thinking about what feminist constellations in law might look like. A feminist constellations approach to legal history goes some way to outlining not only how we might collectively begin to fashion these, but why this is an important endeavour for feminist legal historians to undertake. It seeks to bring feminist and non-feminist, prominent and little-known, and direct and indirect influences together, grounded in a belief that a history that better narrates the feminist endeavour across and within time and space is not only possible, but essential.^151^ While feminist legal historians might already practice this in their scholarship, a feminist constellations approach systematically *requires* us to challenge dominant legal and feminist narratives around whiteness, class and imperialism in new ways.^152^ In so doing, the role of feminism (alongside a feminist approach) is brought into the mainstream of law teaching and scholarship. And while views on how we might do this will differ, the process of identifying feminist constellations in law provides for a better understanding of how the campaigns, organisations and life stories of women can unsettle and challenge previously held assumptions about histories of law, while creating new possibilities for building better and more effective strategies for the future.

## Footnotes

1 ‘Constellation, N., Sense 3’ in *Oxford English Dictionary* (OUP 2023).

2 Suzanne Moore and others, ‘How the Greenham Common Protest Changed Lives: “We Danced on Top of the Nuclear Silos”’ *The Guardian* (20 March 2017).

3 See eg Rosemary Auchmuty and Erika Rackley, ‘Feminist Legal Biography: A Model for *All* Legal Life Stories (2020) 41(2) Journal of Legal History 186; Liz Stanley, *The Auto/biographical I: The Theory and Practice of Feminist Auto/biography* (Manchester UP 1992) 9.

4 Erika Rackley and Rosemary Auchmuty, ‘The Case for Feminist Legal History’ (2020) 40 OJLS 878, 888–9.

5 See also Tracy A Thomas and Tracey Jean Boisseau (eds), *Feminist Legal History: Essays on Women and Law* (New York UP 2011); Joan Wallach Scott, *The Fantasy of Feminist History* (Duke UP 2011).

6 See eg Jennifer Aston and Olive Anderson, *Deserted Wives and Economic Divorce in 19th-Century England and Wales: ‘For Wives Alone’* (Hart Publishing 2024); Caroline Derry, *Lesbianism and the Criminal Law: Three Centuries of Legal Regulation in England and Wales* (Palgrave Macmillan 2020); Erika Rackley and Rosemary Auchmuty (eds), *Women’s Legal Landmarks: Celebrating the History of Women in Law in the UK and Ireland* (Hart Publishing 2018); Russell Sandberg, *Subversive Legal History: A Manifesto for the Future of Legal Education* (Routledge 2023) ch 4; Sharon Thompson, *Quiet Revolutionaries: The Married Women’s Association and Family Law* (Hart Publishing 2022).

7 Though see Rackley and Auchmuty, ‘The Case for Feminist Legal History’ (n 4); Sandra Duffy, ‘Postcolonial Dynamics in Pro- and Anti-Trans Activism in the UK and Ireland’ (feminists@law 2023); Vanessa Munro, *Law and Politics at the Perimeter: Re-evaluating Key Debates in Feminist Theory* (Hart Publishing 2007); Joanne Conaghan, *Law and Gender* (OUP 2013).

8 Our approach brings together feminist legal historical methodology (outlined in Rackley and Auchmuty, ‘The Case for Feminist Legal History’ (n 4)), which in turn builds on the methods of feminist legal studies and feminist history (most obviously Katharine Bartlett, ‘Feminist Legal Methods’ (1990) 103 Harv L Rev 829; Sheila Rowbotham, *Hidden from History: 300 Years of Women’s Oppression and the Fight against It* (Pluto 1973)), the insights of the participants in the Women’s Legal Landmarks project and arguments for micro- and connected history. See further nn 41–4 below and surrounding text.

9 In deploying the insights of astronomy as a means of identifying and disrupting the imaginative hold of particular understandings, methods and narratives on and of legal history, we are building on long-standing arguments of those who have sought to harness the (legal) imagination as an site of discursive/political struggle (see eg James Boyd White, *The Legal Imagination* (2nd edn, University of Chicago Press 1985); Ian Ward, *Shakespeare and the Legal Imagination* (Butterworths 1999) 1).

10 As argued by Rackley and Auchmuty, ‘The Case for Feminist Legal History’ (n 4) 879. It may be that the qualification ‘feminist’ here (and, indeed, throughout this article) is unnecessary. All legal history is (or at least has the potential to be) transformative—or, as Russell Sandberg has argued, ‘subversive’ or ‘anarchic’: Sandberg (n 6). However, while this may be true, our purpose is to go beyond potential and aspiration, and, vitally, the legal academy, in order to locate feminist legal history squarely within the contemporary feminist political project.

11 Caroline Derry, ‘Beyond Firsts: Feminist Biography and Early Women Barristers’ in Victoria Barnes, Nora Honkala and Sally Wheeler (eds), *Women, Their Lives, and the Law: Essays in Honour of Rosemary Auchmuty* (Hart Publishing 2023) 43, 58–9.

12 International Astronomical Union (IAU), ‘The Constellations’ <www.iau.org/Iau/Iau/Science/What-we-do/The-Constellations.aspx> accessed 4 March 2026.

13 The name of the constellation refers to the pattern and area of sky covered by that constellation. Not all stars form the shape or pattern of an officially defined constellation. However, every star falls within the ‘boundary’ of an official constellation and is part of that constellation alone.

14 See eg the ancient Chinese star charts eg Constellations and Myths, Hong Kong space museum <https://hk.space.museum/en/web/spm/resources/teachers-corner/constellations-and-myths/chinese-starlore.html> (accessed 4 March 2026) and the dark constellations identified by Indigenous groups in pre-Hispanic Latin America: Santiago Flórez, ‘From Dark Constellations to Solar Observatories: How pre-Hispanic America Contemplated the Cosmos’ *El País* (22 May 2024). More generally, see Edward Brooke-Hitching, *The Sky Atlas* (Simon & Schuster 2019).

15 Annette S Lee, ‘Anchored to Earth by Starlight: Decolonizing Relationship to Sky’ (2023) 27 Culture and Cosmos 91. Lee’s paper explores an exhibition of collaborative creation of Indigenous Star Maps in Minnesota 2022. It features two Indigenous star maps, Ojibwe Giizhig Anung Masinaaigan (Ojibwe Star Sky Paper) and D(L)akota Makoċe Wiċaŋḣpi Wowapi, created in 2012 as part of the Native Skywatchers Project, which are laid over Greek/ IAU ‘approved’ constellations as a means of offering ‘a subtle translation between cultural cosmologies’ (111). Lee’s paper also reminds us constellations seen in the night sky may be defined and used for different purposes, and that a simplistic reading across from one to the other is neither possible nor helpful. See further <www.nativeskywatchers.com> accessed 4 March 2026.

16 Maggie Aderin-Pocock, *The Art of Stargazing* (Penguin 2023) 2–3.

17 Kirsten Banks, ‘Aboriginal Astronomy Can Teach Us about the Link between Sky and Land’ *The Guardian* (21 May 2018); ‘Milky Way—The Road in the Sky’ <https://americanindian.si.edu/inkaroad/ancestors/creationstories/milky-way.html> accessed 4 March 2026.

18 Steven R Gullberg and others, ‘A Cultural Comparison of the “Dark Constellations” in the Milky Way’ (2020) 23(2) Journal of Astronomical History and Heritage 390.

19 The language of ‘discovery’ here echoes colonial conceptualisations of *terra nullius*: that just as the land being ‘discovered’ by colonisers was not empty, constellations were not brought into existence in the early 1900s. It also reminds us that adopting the language of constellations is not unproblematic. There is a danger in doing so that we perpetuate practices grounded in violence, discrimination and bias. We intend our use of the language of constellations to be inclusive and complicated, allowing for diverse and broad understandings of their form and purpose, while also recognising that our understanding and use is informed by our own prejudice and bias, and as such is inevitably partial. For more on the fiction of *terra nullius* and how this was historically legitimised by law, see Carole Pateman, *Contract and Domination* (Polity 2007) ch 2.

20 ‘Nasa Names Constellations after the Hulk, Godzilla and the Tardis’ *BBC News* (23 October 2018).

21 Valerie Amos and Pratibha Parmar, ‘Challenging Imperial Feminism’ (1984) 17(1) Feminist Review 3.

22 See further Constellation Guide, ‘Asterisms’ <www.constellation-guide.com/asterisms> accessed 4 March 2026.

23 Aderin-Pocock (n 16) 4.

24 Stephen Tonkin, ‘How to Star-Hop the Night Sky’ *BBC Sky at Night Magazine* (5 January 2023).

25 Constellation Guide, ‘Brightest Stars in Constellations’ <www.constellation-guide.com/constellations/brightest-stars-in-constellations> accessed 4 March 2026.

26 Peter Grego, ‘How Many Stars Can a Person See in the Night Sky?’ *BBC Sky at Night Magazine* (11 April 2024).

27 Aderin-Pocock (n 16) 179. ‘Orion’s Belt’ (an asterism within the constellation Orion and the Chinese symbol 白虎（西） (White Tiger (West)) for example, comprises three stars, Mintaka, Alnilam and Alnitak, which are roughly 690 to 2000 light years from Earth and between 50 to over 1000 light years from each other.

28 IAU (n 12).

29 When mapping possible feminist constellations in this article we are not seeking to imply that the women/organisations/locations/events and so on we refer to share an essential or common experience, or a demonstrable connection. Nor are we aiming for completeness. Although we have sought to be expansive in outlining the scope of our approach, our examples remain necessarily partial and limited by our own knowledge. In placing them alongside each other, we are simply seeking to demonstrate the extent to which we can recover relationships that might previously have been overlooked, while gaining a different, and often more nuanced, perspective of historical campaigns, which in turn aid our understanding and response of feminist movements. Nor is the method we outline here applicable only to feminist legal history. Indeed, we hope that the insights emerging from our proposed framework may prove useful in other contexts.

30 The ‘No More Page 3’ campaign ran from 2012 to 2015 in the UK. It successfully convinced the owners of *The Sun* newspaper from publishing topless photos of women on page 3. Jo Cheetham, *Killjoy: Taking on a Macho Media Empire and Winning* (Picador 2023).

31 As Sophie Lewis notes in her introduction to *Enemy Feminisms: TERFs, Policewomen, and Girlbosses Against Liberation* (Haymarket Books 2025) 6-7: ‘I know it can be upsetting to learn that feminisms have been eugenic, colonial, deadly to indigenous lifeways, explicitly antiblack, knowingly dangerous to sex works, violent to queer and feminine people … Women *are* horrible, quite often. Feminists, even, are part of the problem.’

32 Caxton Hall was, among other things, the site of the WSPU’s Women’s Parliament in 1907, and the venue for the first public meeting of the Homosexual Law Reform Society in 1960 and the formation of the National Front in 1967. Sir Michael O’Dwyer, an Irish colonial officer in the Indian Civil Service, was assassinated by Indian revolutionist Sardar Udham Singh while speaking at an event at Caxton Hall in 1940.

33 Miss Bebb, the eponymous heroine of *Bebb v Law Society* [1914], for example, might appear in a constellation on women’s campaigns to enter the legal profession (*Bebb* was a test case brought by four women seeking to become solicitors), early women students at Oxford (she was the seventh woman to study law at Oxford), the sweated industries (she worked as an Investigating Officer for the Board of Trade bringing prosecutors against employers in the sweated trades) and—sadly—20th-century maternal mortality rates, reflecting the fact the she died two months after becoming ill following the birth of her second child). Rosemary Auchmuty, ‘Whatever Happened to Miss Bebb? *Bebb v The Law Society* and Women’s Legal History’ (2018) 31 LS 199.

34 La Marcha de las Mariposas (the March of the Butterflies) takes place on 25 November across Latin America to protest high levels of femicide and violence against women. Its name derives from the Mirabal sisters, who were nicknamed ‘the butterflies’ and fought against Trujillo’s dictatorial regime in 1960s Dominican Republic. Three of the four sisters died in the resistance. Cecelia D’Arville, ‘The Politics of Womanhood: The Mirabal Sister’s Resistance’ (2020) 1 MUNDI 1.

35 At Greenham Common, women sought to juxtapose the violence of the missiles constructed there with the non-violence they sought to preserve, and in doing so organised a teddy bears’ picnic at the base in April 1983. A group of women dressed up as teddy bears, pandas, a jester, a witch and a hot-pink rabbit. Gwyn Kirk, ‘Our Greenham Common: Feminism and Nonviolence’ in Alison M Jaggar, *Living with Contradictions: Controversies in Feminist Social Ethics* (Routledge 1994) 610–19.

36 Lady Hale was the first women appointed to the UK Supreme Court and was its president between 2017 and 2020. In 2019, she read out the Court’s decision in *R (Miller) v the Prime Minister* [2019] UKSC 41, which held that the Prime Minister, Boris Johnson, had acted unlawfully when he advised the Queen to suspend Parliament. Her decision to wear a spider broach on her shoulder was widely discussed in the press. See eg Lauren Cochrane and Martin Belam. ‘Say It with a Brooch: What Message Was Lady Hale’s Spider Sending?’ *The Guardian* (24 September 2019).

37 Ford Cortinas were produced at the Ford Motor Company Limited’s Dagenham plant. A strike by women machinists in 1968 impacted the availability of the Ford Cortina Mk III and is widely recognised as leading to the introduction of the Equal Pay Act in 1970. See further Dawn Watkins, ‘Dagenham Car Plant Strike, 1968’ in Rackley and Auchmuty, *Women’s Legal Landmarks* (n 6) 291.

38 Sandberg (n 6) 27.

39 John McLaren, ‘The Legal Historian, Masochist or Missionary? A Canadian’s Reflection’ (1994) 5 Legal Education Review 67, 78, 81.

40 Elizabeth Grosz, ‘The Untimeliness of Feminist Theory’ (2010) 18(1) NORA—Nordic Journal of Feminist and Gender Research 48, 49.

41 Alexandra Shepard and Garthine Walker (eds), *Gender and Change: Agency, Chronology and Periodisation* (Wiley 2009).

42 See eg the use of the metaphor of jazz music in Elsa Barkley Brown, ‘What Has Happened Here: The Politics of Difference in Women’s History and Feminist Politics’ (1992) 18(2) Feminist Studies 295.

43 An example of this approach can be seen in Sanjam Ahluwalia’s work demonstrating connections among the birth control movements in India, Britain and the United States: Sanjam Ahluwalia, ‘Rethinking Boundaries: Feminism and (Inter)nationalism in Early Twentieth-Century India’ (2003) 14(4) Journal of Women’s History 187.

44 Caitriona Beaumont, ‘The “Silver Thread”: Hazel Hunkins-Hallinan (1890–1982), the Six Point Group, and New Understandings of Intergenerational Female Activism in England, 1960s to 1980’ (2025) 34(4) Women’s History Review 603.

45 Julie Savage, ‘Making History: The Suffrage Movement in *Spare Rib* Magazine from 1972 to 1978’ (2019) 62 French Journal of English Studies 135 [34].

46 Conaghan (n 7); see also Peter Fitzpatrick, ‘Imperial Ends’ in Amy Swiffen and Joshua Nichols (eds), *The Ends of History: Questioning the Stakes of Historical Reason* (Routledge 2012)

47 Conaghan (n 7).

48 See eg Derry, *Lesbianism and the Criminal Law* (n 6) ch 1 and references therein; Valerie Bryson, *Gender and the Politics of Time: Feminist Theory and Contemporary Debates* (Policy Press 2007); Conaghan (n 7) ch 4; Kathryn McNeilly, ‘Are Rights Out of Time? International Human Rights Law, Temporality, and Radical Social Change’ (2019) 28(6) Social and Legal Studies 817.

49 Derry, ‘Beyond Firsts’ (n 11) 55.

50 Emily Grabham, *Brewing Legal Times: Things, Forms and the Enactment of Law* (University of Toronto Press 2016) 13.

51 Derry, ‘Beyond Firsts’ (n 11) 58–9.

52 Writing about the importance of periodisation, Boydston notes that it permits us ‘to hold steady the otherwise constant flurry of difference and change’: Jeanne Boydston, ‘Gender as a Question of Historical Analysis’ (2008) 20(3) Gender History 558, 560. See also Sandberg (n 6).

53 Derry, ‘Beyond Firsts’ (n 11) 47; Allegra Fryxell, Anna Gutgartsb and Oded Y Steinberg, ‘Lost in Time: Periodization and Temporality in Abnormal Times’ (2023) 8(5) Global Intellectual History 549.

54 Tessa Boase, *Mrs Pankhurst’s Purple Feather* (Aurum Press 2018); Robin McKie, ‘A Feather in Their Cap: RSPB’s Victory that Saved Millions of Birds’ *The Observer* (27 June 2021).

55 Grace Woods, ‘Women Reclaim the Night 47 years after First March’ *BBC News* (28 October 2024).

56 Shilpa Phadke, Sameera Khan and Shilpa Ranade, *Why Loiter? Women and Risk on Mumbai Streets* (Penguin Books 2011).

57 A collective of desi feminists seeking to start a conversation around women’s negotiation of public spaces in Pakistan <https://girlsatdhabas.wordpress.com> accessed 4 March 2026.

58 An advocacy group for women in urbanism based in Aotearoa <www.womeninurbanism.co.nz> accessed 4 March 2026.

59 Philippa Gregory, *Normal Women* (William Collins) 73.

60 See Thompson, *Quiet Revolutionaries* (n 6).

61 See further Ann Mumford, ‘Section 32 of the Finance Act 1998’ in Rackley and Auchmuty, *Women’s Legal Landmarks* (n 6) 409.

62 This traditional, Dickensian-like narrative centres on the finding of success and recognition in the world despite adversity. In fictional accounts, this genre is contiguous to bildungsroman or coming of age accounts that often involve movement across social class.

63 Grosz (n 40).

64 See eg Carol Howells, ‘Cyfraith Hywel (The Laws of Hywel Dda), c. 940’ in Rackley and Auchmuty, *Women’s Legal Landmarks* (n 6) 25.

65 See eg ‘Votes for Victorian Women’ *BBC Radio 4* (18 March 2013); Sarah Richardson, ‘The Victorian Female Franchise’ *The Victorian Commons* (18 March 2013).

66 See eg K Crosby, ‘Restricting the Juror Franchise in 1920s England and Wales’ (2019) 37 LHR 163; Erika Rackley, ‘A Short History of Judicial Diversity’ (2023) 76 CLP 265.

67 Phillipa Gregory notes that in the early 1300s men and women labours earned the same wage: employers paid ‘for the skill, not the sex of the worker’: Gregory (n 59) 30. Certainly, in the mid-1200s the mean daily wage for men and women was very close: 1.32 vs 1.06 pence respectively. However, by the mid-1800s, the gap had increased exponentially, with men earning on average at 20.82 pence a day compared to 6.66 pence a day for women. Jane Humphries and Jacob Weisdorf, ‘The Wages of Women in England, 1260–1850’ (2015) 75(2) Journal of Economic History 405, 432.

68 Patricia Murphy, *Time Is of the Essence: Temporality, Gender, and the New Woman* (State University of New York Press 2001).

69 We are grateful to the historian Danaya C Wright for this insight.

70 McNeilly (n 48).

71 See Eldridge’s work exploring the study of the medieval village community in early historical works in the 19th century through to current research: Lorren Eldridge, *Law and the Medieval Village Community Reinvigorating Historical Jurisprudence* (Routledge 2023).

72 Emily Callaci, *Wages for Housework: The Story of a Movement, an Idea, a Promise* (Penguin 2025) xiv. See also Silvia Federici, *Wages for Housework* (Power of Women Collective and the Falling Wall Press 1975).

73 Louise Toupin, *The History of Wages for Housework: A History of an International Feminist Movement, 1972–77* (Pluto Press 2018).

74 Thompson, *Quiet Revolutionaries* (n 6) 21, 28–30.

75 Charlotte Perkins Gilman, *A Study of the Economic Relation Between Men and Women as a Factor in Social Evolution* (Small, Maynard, & Company 1898) 14.

76 ‘Notes for Women’ *Gloucester Citizen* (29 July 1907).

77 ‘Notes for Women’ *Gloucester Citizen* (5 August 1907).

78 Guy Bolton, *Chicken Feed: Or, Wages for Wives. A Comedy in Three Acts* (Samuel French 1924), presented by John Golden.

79 ‘London Letter’ *Hull Daily Mail* (10 September 1929).

80 In emphasising capitalism’s reliance upon the domestic sphere and adopting an anti-work stance, the movement posited that the ‘first step’ to ending this work is ‘to put a price tag on it’: Silvia Federici, *Revolution at Point Zero: Housework, Reproduction, and Feminist Struggle* (PM Press 2012) 37.

81 Who suggested ‘A woman must have money and a room of her own’: Virgina Woolf, *A Room of One’s Own* (Penguin 2004) 4.

82 Women and girls across the world carry out ‘12.5 billion hours of unpaid care work every day’, representing ‘a contribution to the global economy of at least $10.8 trillion a year, more than three times the size of the global tech industry’: Oxfam International, ‘Not All Gaps Are Created Equal: The True Value of Care Work’ <www.oxfam.org/en/not-all-gaps-are-created-equal-true-value-care-work> accessed 4 March 2026.

83 Prabha Kotiswaran, ‘Laws of Social Reproduction’ (2023) 19 Annual Review of Law and Social Science 145, 148.

84 This section draws on the arguments of Sharon Thompson, ‘The Inconspicuous Impact of Feminist Pressure through Law’ (2025) 78(1) Current Legal Problems 29.

85 AR Cleveland, *Woman Under the English Law* (Hurst & Blackett 1896) 298.

86 ibid.

87 Rackley and Auchmuty, ‘The Case for Feminist Legal History’ (n 4) 885.

88 ‘Bow Match Women’s Strike’, Westminster Hall debate 8 October 2013, vol 568, col 22WH.

89 ‘Rights of Women’, HC Deb 3 August 1832, vol 14, col 1086; UK Parliament, ‘Orator Hunt and the First Suffrage Petition 1832’ <www.parliament.uk/about/living-heritage/transformingsociety/electionsvoting/womenvote/parliamentary-collections/1866-suffrage-petition/the-first-petition/> accessed 4 March 2026.

90 Ruth Lamont, ‘Gaol Act 1823’ in Rackley and Auchmuty, *Women’s Legal Landmarks* (n 6) 41.

91 See also Otto Kahn-Freud, ‘Injustices and Inconsistencies in the Law of Husband and Wife’ (1952) 15 MLR 135; AH Manchester, *Modern Legal History 1750–1950* (Butterworths 1980); JC Wood, ‘Attachment of Wage’ (1963) 26 MLR 51, 51 71.

92 Felice Batlan, ‘Legal History and the Politics of Inclusion’ (2014) 26 Journal of Women’s History 155, 155.

93 Thompson, ‘The Inconspicuous Impact’ (n 84).

94 ibid.

95 Krista Cowman, *Women in British Politics, c.1689–1979* (Bloomsbury 2010) 5.

96 Lena Jeger, ‘Power in Our Hands’ in H Hunkins-Hallinan (ed), *In Her Own Right* (Harrap 1968) 148.

97 Pragna Patel, ‘Forced Marriage (Civil Protection) Act 2007’ in Rackley and Auchmuty, *Women’s Legal Landmarks* (n 6) 547.

98 Erika Rackley, Fiona Vera Grey and Sarah Green talking to Elizabeth Woodcraft, ‘Rape Pornography—Winning a Change in the Law’ (*Pod Academy*, 17 August 2015) <https://podacademy.org/podcasts/rape-pornography/> accessed 4 March 2026.

99 Thompson, ‘The Inconspicuous Impact’ (n 84).

100 Anne Morris, ‘Report of the War Cabinet Committee on Women in Industry, 1919’ in Rosemary Auchmuty, Erika Rackley and Mari Takayanagi (eds), *Women’s Legal Landmarks in the Interwar Years: Not for Want of Trying* (Bloomsbury 2024) 49; Harriet Samuels, ‘*Short v Poole Corporation* (1926)’ in Auchmuty, Rackley and Takayanagi (idem) 165.

101 Sandra Stanley Holton, ‘Elmy, Elizabeth Clarke Wolstenholme’ in *Oxford Dictionary of National Biography* (12 November 2020).

102 Mari Takayanagi, ‘Representation of the People Act 1918’ in Rackley and Auchmuty, *Women’s Legal Landmarks* (n 6) 113; June Hannam, ‘International Dimensions of Women’s Suffrage: “At the Crossroads of Several Interlocking Identities”’ (2005) 14 Women’s History Review 543.

103 Thompson, *Quiet Revolutionaries* (n 6) 228.

104 Nicola Godden-Rasul, ‘Section 1 of the Sexual Offences Act 2003’ in Rackley and Auchmuty, *Women’s Legal Landmarks* (n 6) 519.

105 EP Thompson, *The Making of the English Working Class* (Victor Gollancz 1963), cited in David Sugarman, ‘From Legal Biography to Legal Life Writing: Broadening Conceptions of Legal History and Socio-legal Scholarship’ (2015) 42(1) Journal of Law and Society 7, 22.

106 Stuart Banner, ‘Review: *Legal History, Inside and Out*’ (2011) 68 William and Mary Quarterly 725, 728.

107 Máiréad Enright, Kathryn McNeilly and Fiona de Londras, ‘Abortion Activism, Legal Change, and Taking Feminist Law Work Seriously’ (2020) 71 NILQ 359, 385.

108 See further Katharine Cockin, Glenda Norquay and Sowon S Park (eds), *Women’s Suffrage Literature, Volume III: Suffrage Drama* (Routledge 2007). Despite greater ambivalence towards heroines among second-wave feminists, their iconography was still occasionally deployed: the 1979 reprint of Strachey’s *The Cause*, for example, features portraits of Elizabeth Garrett Anderson, Florence Nightingale, Josephine Butler and Millicent Fawcett on its front cover: Ray Strachey, *‘The Cause’: A Short History of the Women’s Movement in Great Britain* (G Bell & Sons 1928).

109 See eg Rackley and Auchmuty, ‘The Case for Feminist Legal History’ (n 4) 897–900; Caroline Derry, ‘Ethel Bright Ashford: More and Less Than a Role Model’ (2020) 29 Women’s History Review 615; Anna Leszkiewicz, ‘Why Is Publishing Suddenly Obsessed with “Rebel” Women?’ [2018] New Statesman. But see Katie Pickles, *Heroines in History: A Thousand Faces* (Routledge 2023) 1, where Pickles seeks to ‘move beyond viewing heroines as women with a singular story whose appearance is random, seeming isolated and out in the cold as individuals. [And] [i]nstead ... gather[s] up and mak[es] broad connections between heroines across time and cultures.’

110 We also play into the hands of the patriarchy. After all, as Rowbotham notes, ‘Great individuals are always easier to contain than a political and social movement’: Sheila Rowbotham, ‘Great Individuals Are Always Easier to Contain than a Political and Social Movement’ *Spare Rib* (24 June 1974) 16, cited in Savage (n 45) [17].

111 See eg Nicola Lacey, *A Life of HLA Hart: The Nightmare and the Noble Dream* (OUP 2006); June Purvis, *Emmeline Pankhurst: A Biography* (Routledge 2002); Barbara Caine, *Destined to Be Wives: The Sisters of Beatrice Webb* (OUP 1986).

112 Pickles (n 109) 5.

113 ‘Margaret Thatcher Tops Woman’s Hour Power List’ *BBC News* (14 December 2016).

114 ‘Jayaben Desai and the Grunwick Strike | Q & A with Historian Dr Shirin Hirsch’ (*People’s History Museum*, 1 April 2022) <https://phm.org.uk/blogposts/jayaben-desai-grunwick-strike/>.

115 Matthew Lyons, ‘The Grunwick Dispute Begins’ *History Today* (8 August 2022). See also Sundari Anitha and Ruth Pearson, ‘The Grunwick Dispute’ (*Striking Women Striking Out*, nd) <www.striking-women.org/module/striking-out/grunwick-dispute> accessed 4 March 2026.

116 Sundari Anitha, Ruth Pearson and Linda McDowell, ‘From Grunwick to Gate Gourmet: South Asian Women’s Industrial Activism and the Role of Trade Unions’ (2018) 23 XXIII-1 Revue Française de Civilisation Britannique 1.

117 ‘10 Week Strike Brings Victory’ (*Striking Women Striking Out*, nd) <www.striking-women.org/sites/default/files/Supreme%20Quilting%20TGWU%20Record.jpg> accssed 4 March 2026.

118 See eg Gallery One and Gallery Two at the People’s History Museum, which places the match workers’ strike in 1888 alongside the Grunwick strike <https://phm.org.uk/digital-experiences-at-phm/> accessed 4 March 2026.

119 Jacqui Tuner, ‘Nancy Astor, First Woman to Take Her Seat in the UK Parliament, 1919’ in Auchmuty, Rackley and Takayanagi (n 100) 41.

120 See eg Emily Rhodes, ‘Women as Child Carers: Arranging and Compensating Mothering in Early Modern Lancashire’ (2025) 30 The History of the Family 108.

121 Jennifer Aston, ‘Petitions to the Court for Divorce and Matrimonial Causes: A New Methodological Approach to the History of Divorce, 1857–1923’ (2022) 43 Journal of Legal History 161.

122 Amos and Parmar (n 21).

123 ibid.

124 ibid.

125 See eg Homer’s description of Odysseus’ journey to the Phaeacian’s country guided by the Pleiads, the Great Bear and Orion the Hunter: Homer, *The Odyssey* (Penguin 2003) 5.270–80.

126 Tory Shepherd, ‘Space Warfare: How the Military Could Be Forced to Give Up GPS and Return to Navigating by the Stars’ *The Guardian* (31 May 2024).

127 For more information on how the sky classroom enables students to explore ‘how interactions with our more-than-human relations shape our understanding of legal obligations’ see University of Victoria ‘A new home for Indigenous legal resurgence’ < https://www.uvic.ca/law/faculty-and-research/centres-and-collaborations/new-indigenous-law-wing.php> accessed 4 March 2026.

128 Chandra Talpade Mohanty, *Feminism Without Borders: Decolonising Theory, Practicing Solidarity* (Duke UP 2003) ch 9.

129 See eg Strachey (n 108), which Juanita Frances referred to as a ‘call to arms’ to continue redressing women’s oppression under the law: Brian Harrison interview with Juanita Frances (LSE, 14 November 1974) 8SUF/B/022.

130 Krista Cowman, ‘“Carrying on a Long Tradition’: Second-Wave Presentations of First-Wave Feminism in *Spare Rib* c. 1972–80’ (2010) 17(3) European Journal of Women’s Studies 193, 194.

131 ibid, quoting Joan Scott, *Gender and the Politics of History* (Columbia UP 1999).

132 ibid, quoting Woolf (n 81) 42.

133 See eg Germaine Greer’s deliberate invoking of the ‘old suffragettes’ on the first page of *The Female Eunuch* (Bantum 1970). See also Dale Spender, *There’s Always Been a Women’s Movement This Century* (London 1983) 6; Strachey (n 108).

134 Savage (n 45) [37].

135 The suffragette moniker has also been applied to climate change activists. See eg Helen Pankhurst, ‘My Suffragette Grandmothers Are Now Seen as Heroes. Today’s Climate Protesters Will Be Too’ *The Guardian* (15 November 2022); Lanra Bakare, ‘Climate Activists Who Target Artworks “Using Suffragette Tactics”, Says Artist’ *The Guardian* (14 January 2025). These references are often (though not always) one-dimensional, lacking in nuance and divorced, whether by ignorance or design, from the darker underbelly of the history they are seeking to invoke. Our purpose here is not to assess the validity or otherwise of their claims; rather, it is simply to point out *that* they do so, to use this as an illustration of the currency and timelines of history within feminist campaigns, and the extent to which these campaigns see—and present—themselves within existing feminist constellations.

136 Fiona Macdonald, ‘Pussy Riot, Femen and SlutWalk Are Working Hard to Give Feminism a Voice’ *Metro* (4 June 2013).

137 Sam Wilson, ‘Ukraine’s Femen: Topless Protests “Help Feminist Cause”’ *BBC News* (24 October 2012).

138 The London Society for Women’s Suffrage was renamed ‘The Fawcett Society’ in honour of Milicent Fawcett in 1953.

139 Aasma Day, ‘WASPI Women Will Keep on Fighting—We Owe It to the Suffragettes’ *The i paper* (17 March 2025).

140 See eg Sarah Pedersen, ‘“It’s What the Suﬀragettes Would Have Wanted”: The Construction of the Suﬀragists and Suﬀragettes on Mumsnet’ (2023) 23 Feminist Media Studies 1543; Duffy n 7; For Women Scotland, ‘Suffragette Scarf Giveaway’ (19 January 2024) <https://forwomen.scot/19/01/2024/suffragette-scarf-giveaway/> accessed 4 March 2026; Day (n 139).

141 ‘The Countess Didn’t Fight for This (TCDFFT)’ <www.thecountess.ie/> accessed 4 March 2026.

142 Pedersen (n 140) 1556.

143 Laura Lee Downs, *Writing Gender History* (2nd edn, Bloomsbury 2010) 22.

144 Sandra Berns and Paula Baron, ‘Bloody Bones: A Legal Ghost Story and Entertainment in Two Voices—To Speak as a Judge’ (1994) 2 Australian Feminist Law Journal 125, 127.

145 In the same way as Cowman describes *Spare Rib* as positioning ‘itself historically within a “long tradition’” of feminist magazines, citing as its ‘forerunners’ titles associated with first-wave feminism such as *Shafts* and *Votes for Women*, which simultaneously informed their readership while attempting to motivate them into activism. Spare Rib Collective, ‘Fifty Years of Women’s Suffrage’ (editorial) *Spare Rib* 73, August 1978 in Cowman (n 130) 197.

146 An example of this is Jess Philips MP’s reading the names of every woman killed by men over the previous year on International Women’s Day, drawing on the academic and campaigning work of Femicide Census, which collates the list of the women and girls aged 14 and above who have allegedly been killed or are known to have been killed by men in the past year, to ensure the women’s names are formally recorded in the Parliamentary record. Most recently: ‘International Women’s Day’ HC Deb 6 March 2025, vol 763, col 520.

147 Enright, McNeilly and de Londras (n 107) 385.

148 ‘Going to Space Reset My Priorities in Life’ *BBC 5 Live* (10 November 2025) <www.bbc.co.uk/news/videos/c04g2yx9nk1o> accessed 4 March 2026.

149 Claire Cock-Starkey, *Lore of the Stars: Folklore and Wisdom from the Skies Above* (Quarto Publishing 2023).

150 Jessica Sequeira, ‘Connected History: Essays and Arguments’ (2023) 46 South Asia: Journal of South Asian Studies 254, 257.

151 For more on the application of our methodology, see Sharon Thompson and Erika Rackley, ‘A Feminist Star Atlas: Mapping Legal History through Feminist Constellations’ in Rosemary Auchmuty, Caroline Derry and Danaya Wright (eds), *Research Handbook on Gender, History and Law* (Edward Elgar forthcoming).

152 Amos and Parmar (n 21); Mrinalini Sinha, ‘Gender and Nation’ in Bonnie Smith (ed), *Women’s History in Global Perspective* (University of Illinois Press 2004) 229.

